# Recent Developments and Perspectives of Recycled Poly(ethylene terephthalate)-Based Membranes: A Review

**DOI:** 10.3390/membranes12111105

**Published:** 2022-11-05

**Authors:** Kirill Kirshanov, Roman Toms, Gadir Aliev, Alina Naumova, Pavel Melnikov, Alexander Gervald

**Affiliations:** M.V. Lomonosov Institute of Fine Chemical Technologies, MIREA—Russian Technological University, 119571 Moscow, Russia

**Keywords:** poly(ethylene terephthalate) PET, chemical recycling, unsaturated polyester resins, track-etched membranes, ion track technology, nanofibrous membranes, electrospinning, phase inversion membranes, phase separation

## Abstract

Post-consumer poly(ethylene terephthalate) (PET) waste disposal is an important task of modern industry, and the development of new PET-based value added products and methods for their production is one of the ways to solve it. Membranes for various purposes, in this regard are such products. The aim of the review, on the one hand, is to systematize the known methods of processing PET and copolyesters, highlighting their advantages and disadvantages and, on the other hand, to show what valuable membrane products could be obtained, and in what areas of the economy they can be used. Among the various approaches to the processing of PET waste, we single out chemical methods as having the greatest promise. They are divided into two large categories: (1) aimed at obtaining polyethylene terephthalate, similar in properties to the primary one, and (2) aimed at obtaining copolyesters. It is shown that among the former, glycolysis has the greatest potential, and among the latter, destruction followed by copolycondensation and interchain exchange with other polyesters, have the greatest prospects. Next, the key technologies for obtaining membranes, based on polyethylene terephthalate and copolyesters are considered: (1) ion track technology, (2) electrospinning, and (3) non-solvent induced phase separation. The methods for the additional modification of membranes to impart hydrophobicity, hydrophilicity, selective transmission of various substances, and other properties are also given. In each case, examples of the use are considered, including gas purification, water filtration, medical and food industry use, analytical and others. Promising directions for further research are highlighted, both in obtaining recycled PET-based materials, and in post-processing and modification methods.

## 1. Introduction

Poly(ethylene terephthalate) waste mismanagement causes significant environmental damage [1]. Poly(ethylene terephthalate) is a large tonnage polyester, with its waste at about 12% of the total solid waste. The range of products, based on PET includes mainly packaging and fibers [2].

Packaging usually includes bottles for soft drinks, but films and containers are also common. PET bottles are widely known to be an easily retrievable type of waste. That is why the main poly(ethylene terephthalate) commodity form that is recycled, is post-consumer PET bottles [2]. They can be difficult to manage, due to the presence of other polymer materials, but the lids and labels are easily removable.

The situation is more complicated in the case of fibers [3]. Textile fibers for the production of clothing and synthetic down are relatively easy to handle. A more serious problem is the utilization of polyester tire cord, which is contaminated with hard-to-separate crumb rubber [4,5,6]. Medical waste, represented mainly by used medical dressings, is another source of post-consumer PET [7,8].

There are various approaches to the disposal of poly(ethylene terephthalate) waste. One of them is the reuse of PET products, but this method is not suitable for contaminated products, especially food and medical goods. The main approach that is developed, is recycling. Recycling with a decrease in the added value of the material is called downcycling, with an increase-upcycling [1]. One way to increase the efficiency of global recycling is to modify the labelling for better waste management in each region [9]. However, most of the studies are still focused on recycling methods. Possible solutions to the problem of PET recycling are, on the one hand, the development of applications for recycled products and, on the other hand, the development of new recycling methods.

New applications for recycled PET materials include but are not limited to modified polyester fibers and 3D printing materials [10,11], unsaturated polyester resins [12,13,14], plasticizers [4] and dyes [15]. One of the most promising value-added products, based on PET, are membranes. Poly(ethylene terephthalate) has a number of advantages that determine its applicability for the production of membranes: high physical and mechanical properties, chemical resistance and thermal stability [16]. PET can be processed by all methods of thermoplastic polymers fabrication, and it can be used to produce strong fibers and films of various thicknesses. Membranes, based on poly(ethylene terephthalate) are primarily used for water treatment and gas purification, as well as matrixes filled with various nanomaterials [17,18]. Moreover, PET and other copolyesters possess reactive ester, hydroxyl and acid groups, which make it possible to impart special properties to membranes, due to the chemical modification of surfaces. Thus, the use of recycled poly(ethylene terephthalate) and materials, based on it, in industry is relevant. The processes for obtaining PET-based membranes can be classified as upcycling, due to the increase in added value [13].

The aim of this review is to consider both methods for the recycling of poly(ethylene terephthalate) and the directions for using PET and copolyesters, based on it to obtain membranes. The key advantage of such a comprehensive consideration is the ability to formulate the requirements for recycled PET (rPET) materials, according to their further use in membranes. At the same time, we have highlighted the possibilities for the development of membrane technologies, based on the collected data on the properties of PET-based materials and the possibilities of their production. We have not seen reviews of this kind previously in the literature. Section 2 discusses various recycling methods, especially chemical ones. Both methods for obtaining PET, similar in properties to the primary one, and the main ways for obtaining copolyesters, based on recycled PET, are described. Section 3 represents various methods for obtaining membranes, including track etching, electrospinning, phase separation, and others.

## 2. Poly(ethylene terephthalate) Recycling Methods

**Figure 1:**
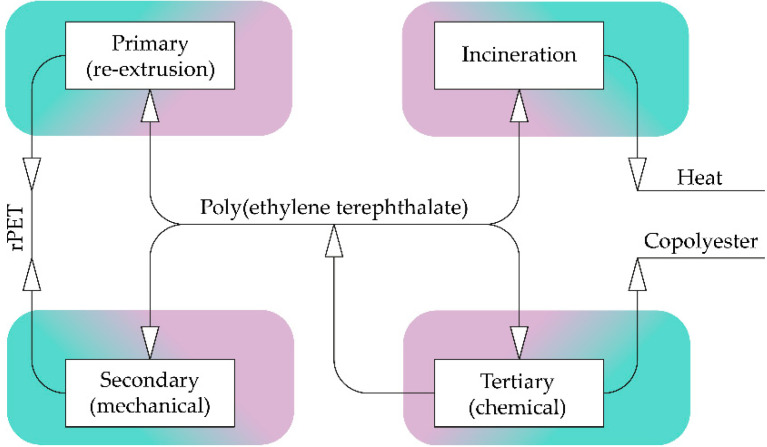
Recycling methods and products.

A large number of studies, in recent years, have been devoted to PET recycling [2,3,19,20,21]. Recycling methods are usually divided into primary, secondary and tertiary recycling [2,19]; incineration is sometimes considered as a quaternary recycling method [2]. The relationship between recycling methods and its products is shown in Figure 1.

Industrial waste that has not yet been contaminated with biological contaminants or impurities of other materials is subjected to primary recycling or re-extrusion. Waste is crushed and returned to the process, along with fresh raw materials in this recycling method.

Secondary recycling, sometimes referred to as mechanical or physical recycling, differs from the primary method in that the feedstock is post-consumer PET. That is why secondary recycling includes the stages of material sorting and thorough cleaning in addition to crushing [2,19,20].

Quaternary recycling is an incineration with the release of thermal energy [2]. This method is often the only one possible for the disposal of heavily contaminated polymer waste. The same group of methods includes pyrolysis, which is used, in particular, for the disposal of polyester tire cord [5].

The product of both primary and secondary recycling is rPET, which is inferior in properties to the primary (synthesized) poly(ethylene terephthalate). Quaternary recycling leads to the formation of heat and combustion products of PET and contaminants. That is why all of these methods are referred to as downcycling [22].

Tertiary recycling (chemical) is devoid of many of the disadvantages of the other methods, so a significant part of researchers’ focus on it. A qualitative comparison of the recycling methods, in terms of the presence (“+”) or absence (“-”) of a number of key advantages or disadvantages, that are important for the value of the resulting processed products and also the impact on the environment is shown in Table 1.

The most common method of processing polymer waste is incineration, but the problem of its ecological impact is currently relevant. Industrial and most clean post-consumer PET waste is processed by primary and secondary recycling. The main problems of these methods are the degradation of polymer properties in each processing cycle and the high sensitivity to moisture [19]. Even a few percent water content results in a significant reduction in the molecular weight. Therefore, the technological process requires the use of dryers and crystallizers to prevent particles from sticking together in the dryers. Furthermore, the inability to extract the dye dictates the separate collection of waste of different colors, which makes the process more expensive. Although chemical recycling processes are devoid of these disadvantages, they have their own problems that hinder them from being implemented in industry.

### 2.1. Chemical Recycling Methods

Chemical recycling methods could be divided into two groups, depending on the desired product: PET, which is similar in properties to the primary one, and copolymers.

The following types of chemical recycling methods are usually distinguished: hydrolysis, alcoholysis, acidolysis, aminolysis, ammonolysis [23,24,25]. It is also possible to attribute the interchain exchange and solid-state polycondensation to chemical methods, since these processes are also based on chemical reactions. The products aminolysis and ammonolysis are not included in this review, since only methods that make it possible to obtain polyesters are considered.

**Figure 2:**
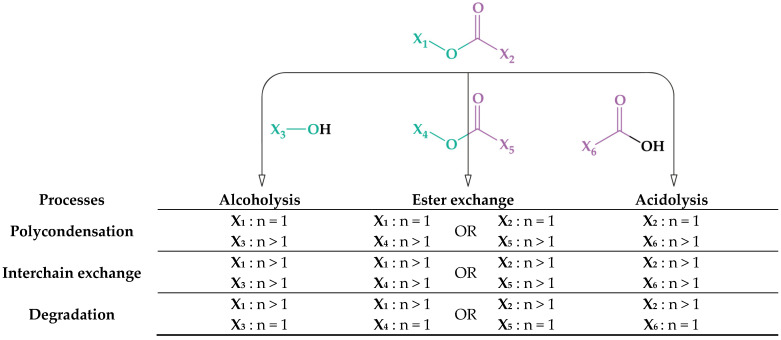
Transesterification-based processes.

Two main groups of methods can be distinguished, depending on the chemical reactions carried out: esterification-hydrolysis-based and transesterification-based. The first group is based on the reactions of the hydroxyl and acid groups with the release of water, which leads to an increase in the molecular weight of the polyester, and the reverse reactions, which lead to its decrease. Polyesterification and hydrolysis processes are included in this group. The group of transesterification-based processes includes reactions, based on the action of hydroxyl and acid groups on the ester group or the exchange interaction of ester groups with each other. This group includes reactions of polytransesterification (polycondensation), alcoholysis, acidolysis. The general scheme of such interactions is shown in Figure 2.

The reaction can lead to an increase in the molecular weight (polycondensation) or its decrease (degradation), depending on the length of the X chain. Interchain exchange reactions do not lead to a change in the number of the average molecular weight of the polyester mixture, but can change the molecular weight distribution.

The system in Figure 2 includes both the reactions that describe the production of poly(ethylene terephthalate) and those that describe the production of copolyesters. It should be mentioned that the contribution of ester exchange reactions under normal conditions of chemical recycling processes is insignificant.

### 2.2. Poly(ethylene terephthalate) Preparation Methods

To obtain poly(ethylene terephthalate), which is similar in properties to the primary one, post-consumer poly(ethylene terephthalate) is depolymerized under the action of various agents (water, methanol, ethanol, ethylene glycol, diethylene glycol, other glycols, oligoethers with hydroxyl end groups), the resulting monomers are purified, and then their subsequent polycondensation is carried out.

Depolymerization is usually carried out at high temperatures, often over 150 °C. Thus, glycolysis is currently the most promising method, since it can be carried out at higher temperatures at an atmospheric pressure than hydrolysis and methanolysis [26]. In addition to the need for equipment that operates at an elevated pressure, these processes are inferior to glycolysis since they require the separation of products: ethylene glycol and terephthalic acid or dimethyl terephthalate. The product of PET glycolysis, bis(2-hydroxyethyl) terephthalate, in turn, is easily purified by recrystallization in water [27]. The focus of research efforts in this area is the study of new catalytic systems.

The most typical transesterification catalysts are zinc compounds [27]. Zinc acetate [28,29,30] and zinc sulfate [31] are widely represented in the literature. Oxides of zinc, manganese, cobalt, aluminum, magnesium, titanium [32,33] and complex compounds [34,35,36] are fairly wide used. Glycolysis catalysis with the aid of nanoparticles and nanotubes is distinguished among the newest directions. There are reports on the use of nanoparticles of iron oxide (III) [37,38], silicon oxide [39] and their mixtures [40], calcium, strontium and barium oxides [41], graphene and manganese oxides [42], aluminum-magnesium hydroxide [43] and cobalt [44]. Both titanium [45,46] and carbon [47] nanotubes are used. is the use of ionic liquids is also well-known [48], including deep eutectic solvents [49,50].

More non-trivial methods of catalysis are also known. For example, it was proposed to use calcium oxide, isolated from eggshells, as a catalyst [51]. The use of urea [52] or enzymes [53] as catalysts has also been proposed. The use of microwave radiation in PET glycolysis, usually with the simultaneous use of a catalyst, is described in detail among the physical effects [34]. Studies using gamma radiation are also known [54].

The process can be organized as both heterogeneous and homogeneous. PET is insoluble in ethylene glycol, so glycolysis is usually carried out with an interface reaction. However, there are methods for the homogeneous glycolysis in solution and in melt. The processes in solution are carried out using an additional solvent, for example, dimethyl terephthalate [29,55], or in a return flow of the resulting bis(2-hydroxyethyl) terephthalate monomer [56]. Melt-based methods are carried out in an autoclave [57] or under the action of oligoesters [4,13]. Such oligoesters should have a concentration of active groups sufficient for a fast reaction, but insufficient for the intensive isolation of a low molecular weight compound (water, alcohol, glycol). They must also be highly compatible with poly(ethylene terephthalate). Both oligo(ethylene terephthalate) [4] and other oligoesters could be used [13].

### 2.3. Copolyester Preparation Methods

**Figure 3:**
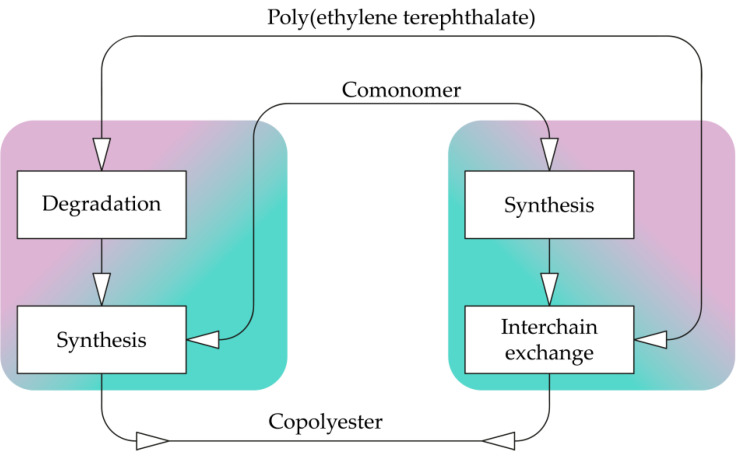
The approaches to the recycling of poly(ethylene terephthalate) into copolyesters.

Two approaches can be applied to the processing of recycled poly(ethylene terephthalate) into copolyesters, which are shown in Figure 3.

The first one consists of the chemical destruction of PET to monomers or oligomers and the further polycondensation with added comonomers. Chemical degradation is carried out in the same way, as described in Section 2.2. Monomers of isophthalate [58] and phthalate [10], 2,5-furandicarbonate [59], di- and polyethylene glycols [60], 1,4-cyclohexanedmethanol [61], 1,2- and 1,3-propanediol [62], 2-methyl-1,3-propanediol [63], tricyclodecanedimethanol [64]. The advantages of the process are flexibility, the ability to combine with primary production and the use of monomer purification, which is much easier, compared to polymer purification. Moreover, such a copolyester can be used for food or medical purposes. However, the cost of such a polymer will be higher, primarily due to chemical recycling. This drawback is fundamental, since secondary PET often exceeds the price of primary PET even without this stage.

The second approach is to obtain polyester from the same comonomers and carry out the interchain exchange reaction with secondary poly(ethylene terephthalate) [10,65,66]. The process proceeds as follows: a small amount of a mixture containing another polyester and additives, such as a dye, is poured into the PET melt, followed by stirring for a certain time. The method is obviously cheaper than the first one. It is noteworthy that the method will allow for the increase in the molecular weight of the recycled polyester, even if the end groups are destroyed and lose activity during the operation.

## 3. Membrane Preparation Methods

The idea of using recycled poly(ethylene terephthalate) to produce membranes has a number of advantages. The use of rPET and materials, based on it, makes it possible to recycle poly(ethylene terephthalate) waste, reduce the global consumption of polymers and reduce the cost of membrane production [17]. The main methods to obtain various membranes from poly(ethylene terephthalate) are ion track technology (Section 3.1), electrospinning (Section 3.2) and phase inversion (separation) (Section 3.3).

### 3.1. Ion Tracking Technology

PET-based track membranes are already a widespread commercial product, and the technologies for their production have been developed in detail. That is why the use of materials, based on post-consumer poly(ethylene terephthalate), is promising.

Track membranes are widely used in industry. They are most often applied for water treatment (membrane distillation, osmosis, ultrafiltration, micro-filtration) [18,67]. Another new direction is the synthesis of magnetic nanotubes and nanowires in the pores of track membranes [68]. A suitable material for producing such nanowires is an alloy of iron and nickel [69,70,71]. Another potential application of the track membranes is their use as separators in lithium-sulfur batteries [72].

The feedstock for producing track membranes is a polyester film. It is irradiated with krypton ions using a cyclotron; argon, xenon [73], helium [74] and other elements are also used. Thus, the initial form of secondary poly(ethylene terephthalate) or a copolymer, based on it to obtain track membranes, should be a film with a thickness of 5 to 24 microns. The films obtained are then treated with UV radiation [75,76]. The duration of the UV irradiation and its application on one or both sides of the film determines the shape of the pores, which affects the ion transport [76]. Membranes with cylindrical [77], conical and double conical [78] channels are common. Chemical etching with an alkali solution is carried out after the UV treatment and the resulting membrane is washed with a neutralizing solution and distilled water. However, Wang et al. [79] showed that track membranes with a high permeability could also be obtained by heat treatment instead of chemical etching.

**Figure 4:**
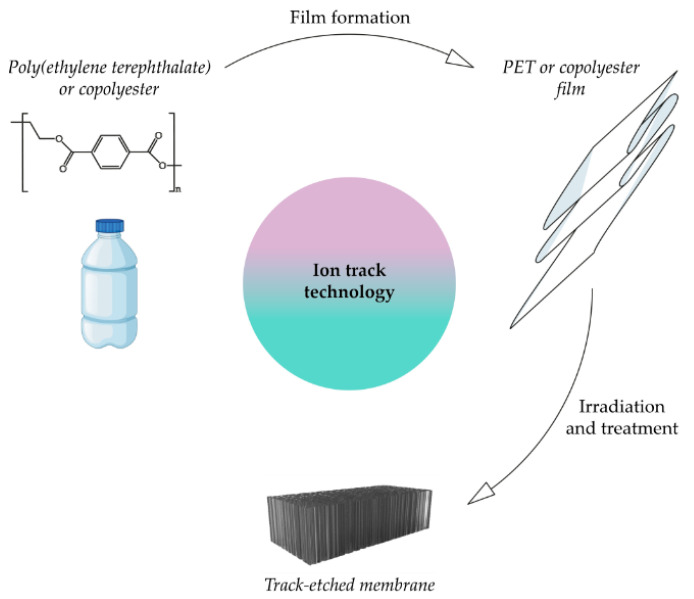
Scheme for obtaining a membrane from recycled poly(ethylene terephthalate) or a PET-based copolymer by ion tracking technology.

**Figure 5:**
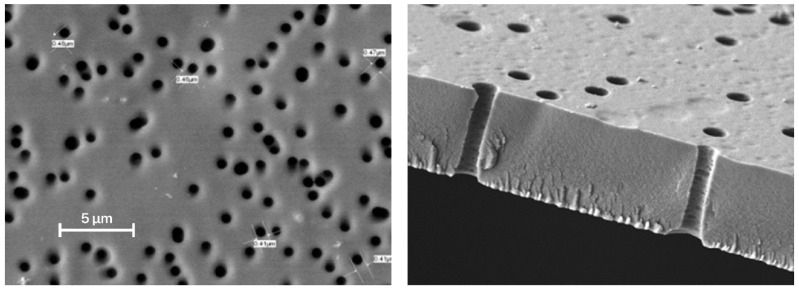
SEM images of PET track-etched membranes [18].

The method for obtaining a track membrane from recycled poly(ethylene terephthalate) or a copolymer, based on it, is shown in Figure 4. SEM images of poly(ethylene terephthalate) track membranes are shown in Figure 5.

Below, we will consider the features of obtaining membranes from PET and copolymers in turn.

#### 3.1.1. PET-Based Track Membranes

To impart special properties to the resulting membranes, they are subjected to post-processing after production. In addition to the thickness of the membrane and the pore sizes, which are adjusted during the production of the membrane, it is possible to adjust the hydrophobicity and hydrophilicity of the membrane surface. Some modification methods, such as graft copolymerization, require special chemical reactive groups.

The main way to impart hydrophobicity to the surface of a track membrane, based on PET, is the grafting of dichlorodimethylsilane or perfluoro-dodecyltrichlorosilane [80], triethoxyvinylsilane [81] and trichloro(octyl)silane [82]. Active sites for the attachment of silanes to the surface of the track membrane are hydroxyl and acid end groups, which are formed during the hydrolysis of ester groups of polyethylene terephthalate, during the chemical etching [82]. Hydrophobic membranes could be used primarily for water treatment. In particular, Korolkov et al. considered the use of such membranes for wastewater treatment and seawater desalination [80], separation of oil–water emulsions is also possible [81]. In the first case, membrane distillation (MD) is performed, which is a thermally driven process of vapors through a non-wettable hydrophobic membrane. MD has advantages, such as a high separation factor, high decontamination factor in only one stage and a low working pressure. Nevertheless, high energy consumption of MD (331,600 kW), in comparison to reverse osmosis (RO) and RO-MD hybrid plant (113,000 kW) as well as the high cost of the membrane used, is key the aspect for MD restrictions in many fields of applications [80,82]. In the case of oil-water emulsions separation, the process is based on filtration, in which water does not pass through the membrane due to the high hydrophobicity of the material, but the organic phase passes [81]. When choosing a membrane, the following characteristics should be taken into account: membrane porosity, membrane pore size distribution, and hydrophilicity/hydrophobicity of the material. However, the choice and efficiency of the separation methods also depend on the properties of the oil-water mixture itself, in particular, the size of the oil droplets, and other factors, such as oil concentration and chemical composition [81].

Hydrophilization methods are systematized in Table 2.

Various monomers containing multiple bonds are grafted onto membranes to modify their properties. Korolkov et al. demonstrated the method of glycidyl methacrylate, acrylonitrile and their mixtures grafting onto a PET membrane [88]. The obtained polymer chains were subjected to further reactions, where the grafted monomers played the role of the precursor for the formation of amino and amidoxime groups on the track membrane. The resulting material is suitable for use in electrochemical sensors. For example, the grafting of acrylic acid and 4-vinylpyridine allows the resulting membranes to act as sensors in the electrochemical determination of heavy metal ions [89]. Nguyen et al. grafted substrates made of poly(methyl methacrylate) to a PET track membrane, using (3-glycidyloxypropyl)trimethoxysilane as a bonding agent [90]. The authors proposed to use the composites obtained by the described method for cell cultivation and drug testing. Parmanbek et al. grafted Poly(2-(Dimethylamino)Ethyl Methacrylate) onto PET membranes for wastewater treatment from the As(III) ions [91]. YanLi et al. obtained a membrane selected to lead ions by grafting enzymes to a track PET membrane [92].

Another way to change the properties of membranes is to obtain a composite track-etched membrane, in particular, by coating the membrane with a layer loaded with nanoparticles or embedded nanotubes or nanowires; examples are given in Table 3. There are many ways to obtain composite membranes [93,94], including a chemical bath or chemical and physical vapor deposition, electrodeposition and electroless plating reactions.

#### 3.1.2. Copolyester-Based Track Membranes

The use of different PET-based copolyesters is another approach to change the properties of the membrane, in addition to the different methods of surface modification, grafting and the application of various components described above. For example, poly(ethylene naphthalate) (PEN) [130,131] and polylactide (PLA) [132] are used to fabricate track membranes. PEN films are less wettable than PET, however the strength properties of the two polymers are similar. The key property of track membranes, based on PLA, is the biodegradability. Thus, varying the ratio of the monomers during copolymerization makes it possible to tune the properties of the resulting material.

Copolymers of terephthalate and naphthalate, as well as terephthalate and lactide, are known. Such copolyesters can be obtained by chemical recycling in both the first and second ways (Figure 3). For example, PET-PEN copolymers are obtained by polycondensation of dimethyl-2,6-naphthalenedicarboxylate and dimethyl terephthalate with ethylene glycol [133] or by interchain exchange of PET and PEN [134,135]. PET-PLA copolymers are obtained by the polymerization of bis(2-hydroxyethyl) terephthalate and oligo-L-lactide [136] or by the interchain exchange of oligoethylene terephthalate and polylactide [137]. Further study of the properties of polyester track membranes made from copolymers, based on polyethylene terephthalate is an important task.

### 3.2. Electrospinning

**Figure 6:**
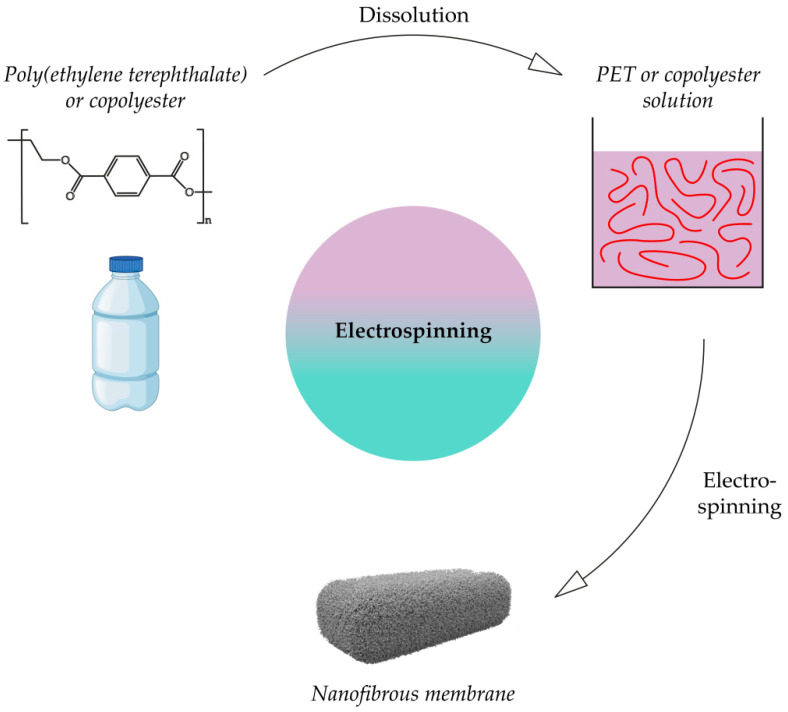
Scheme for obtaining a membrane from rPET or a PET-based copolymer by electrospinning.

**Figure 7:**
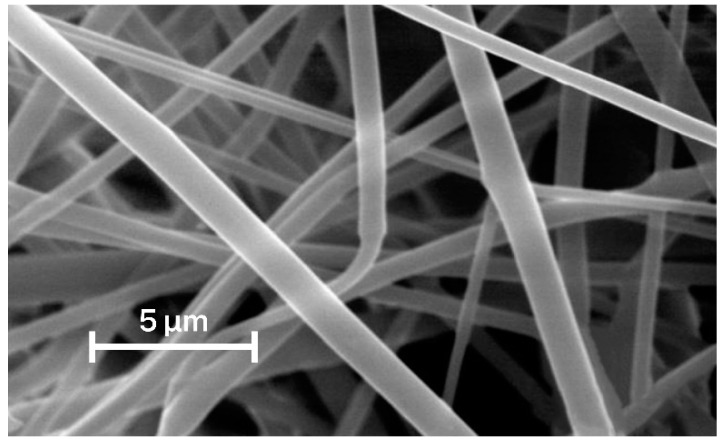
SEM image of a PET nanofibrous membrane [143].

Nanofiber membranes are obtained by electrospinning from a melt and a polymer solution [16,138]. In practice, in the case of PET, the solution molding method is usually used, since the surface tension of the PET melt is high. The scheme for obtaining a nanofibrous membrane from recycled polyethylene terephthalate or copolymers, based on it is shown in Figure 6. Solvents used in the various works are listed in Table 4. An example of a SEM image of poly(ethylene terephthalate) electrospun membrane is shown in Figure 7.

**Table 4:** Solvents used in electrospinning.

Solvent	Ratio, Weight	References
Trifluoroacetic acid	—	[139,140]
Trifluoroacetic acid, dichloromethane	7:3	[140,141,142,143]
1,1,1,3,3,3-Hexafluoro-2-propanol, dichloromethane	2:8	[144,145]

From a technological point of view, the resistance of PET to solvents, which is usually considered an advantage, becomes a disadvantage during electrospinning.

The field of application of nanofiber membranes, based on polyethylene terephthalate obtained by electrospinning [16], is extensive. First, they are used for water treatment by membrane distillation along with track membranes. An important requirement for such membranes is its hydrophobicity, and the surface can be modified, for example, with perfluorodecyltriethoxysilane, to achieve this property [139]. Hydrophobic membranes, based on poly(ethylene terephthalate), modified with organosilicon compounds were used for the membrane distillation [139], as in the work of Korolkov et al. [82]. Noan et al. reported on the hydrophobic nanofiber membranes, based on rPET, coated with polydimethylsiloxane for water-oil separation [141]. Such membranes could be used for water purification from various types of fuels and oils [140]. Nanofiber PET membranes could also be used for air filtration, including filtration from cigarette smoke [142], aerosols and viruses [146], in particular, SARS-CoV-2 [143]. That is why such membranes can be used in personal protective equipment [144]. Electrospun PET-based membranes find a number of more important applications besides water and air purification, for example, as a substrate (matrix) for the production of membranes from other materials by other methods. An example of such a use is the work of Nuanhuan et al., who obtained porous polysulfone membranes by the non-solvent induced phase separation (NIPS) method [147]. The details of the process are discussed in Section 3.3.

#### 3.2.1. PET-Based Nanofibrous Membranes

In addition to perfluorodecyltriethoxysilane [139] and polydimethylsiloxane [141], hydrophobic properties are imparted to electrospun membranes through the addition of modified silica particles. Tas et al. used fluorinated silane functionalized SiO_2_ nanoparticles [148]. Rongkun et al. added thermoplastic polyurethane nanofibers containing silver and SiO_2_ nanoparticles to a membrane, based on PET nanofibers, to achieve electromagnetic shielding [149]. Silver nanoparticles were also added to the PET electrospun membrane, by Grumezescu et al., to impart antimicrobial properties [150]. Other additives for modifying the properties of such membranes are cellulose nanocrystals and castor oil [151] and carbon nanotubes [152]. Cellulose nanocrystals are hydrophilic crystalline rod-like nanocrystals with a high degree of crystallinity. The combined effect of cellulose nanocrystals as a reinforcing material and castor oil as a compatibilizer allowed to increase the strength factor and Young’s modulus by approximately eleven and ten times, respectively, compared to pure rPET [151]. The presence of carbon nanotubes significantly improved the adsorption properties of the material. This is achieved through their high surface area, high porosity, hollow and layered structures, and electrical and hydrophobic interactions with pollutant ions [152].

Unlike track membranes, in which grafting is always carried out on the surface of the resulting membrane, grafted PET nanofibrous membranes could also be obtained in a different way. For example, Gun Gok et al. grafted hydroxyethyl methacrylate onto polyethylene terephthalate fibers, then dissolved them in trifluoroacetic acid followed by electrospinning the resulting solution [153]. This approach makes it possible to obtain nanofibers of a more uniform composition from a graft copolymer than in the case of grafting to the membrane surface.

#### 3.2.2. Copolyester-Based Nanofibrous Membranes

The use of copolyesters, instead of pure PET, has a number of advantages. In addition to improving the performance properties of the membranes, the use of PET-based copolymers that are more soluble in organic solvents makes it possible to reduce the cost of obtaining membranes through the use of common inexpensive solvents, instead of trifluoroacetic acid and 1,1,1,3,3,3-Hexafluoro-2-propanol. Chin-San et al. used environmentally friendly copolyester poly(butylene adipate-co-terephthalate) [154]. This copolymer is biodegradable, due to the adipate units, but it has a number of disadvantages: a high water absorption and a low thermal conductivity. These shortcomings were corrected by the authors by grafting acrylic acid and applying silica airgel powder. Poly(ethylene oxide terephthalate) and poly(butylene terephthalate) copolyesters, used by Danti et al., are soluble in a mixture of chloroform and hexafluoro-2-propanol in a 7:3 volume ratio [155]. The main advantage of poly(ethylene oxide terephthalate)/poly(butylene terephthalate) is its biodegradability, as is the case of polylactide and adipates. Controlling the ratio of poly(butylene terephthalate) rigid segments and poly(ethylene oxide terephthalate) flexible segments, makes it possible to control the mechanical properties of the copolymer and the hydrolysis rate, since ether bonds in polyethylene oxide are not affected by it. For this reason, the polymer is widely used in medicine. Chia-Jung et al. used biodegradable copolyester poly(ethylene sebacate-co-adipate), with 1,3,5-benzenetricarboxylic acid added to improve mechanical properties [156]. The resulting copolymer was dissolved in chloroform, dimethylformamide, and their mixtures in ratios of 2:8, 1:1, and 8:2. This polymer is not only biodegradable, but also bio-based, since sebacic acid is obtained industrially from castor oil, a product of pressing the seeds of the castor bean *Ricinus commúnis*.

It Is known that phthalate and isophthalate, naphthalate, diethylene glycol, propylene glycol and many others co-monomers also increase solubility in organic solvents, as do sebacate, adipate, butylene glycol and polyethylene glycol units. In addition, their introduction into the polyester chain changes it physical, mechanical and thermal properties and chemical stability [10,58,59,60,61,62,63,64,65,66]. Thus, copolyesters that include these units are potentially interesting raw materials for the preparation of nanofibrous PET-based membranes.

### 3.3. Phase Inversion or Separation

The main application of porous membranes obtained by phase inversion (phase separation) is water treatment [157], commonly ultrafiltration.

**Figure 8:**
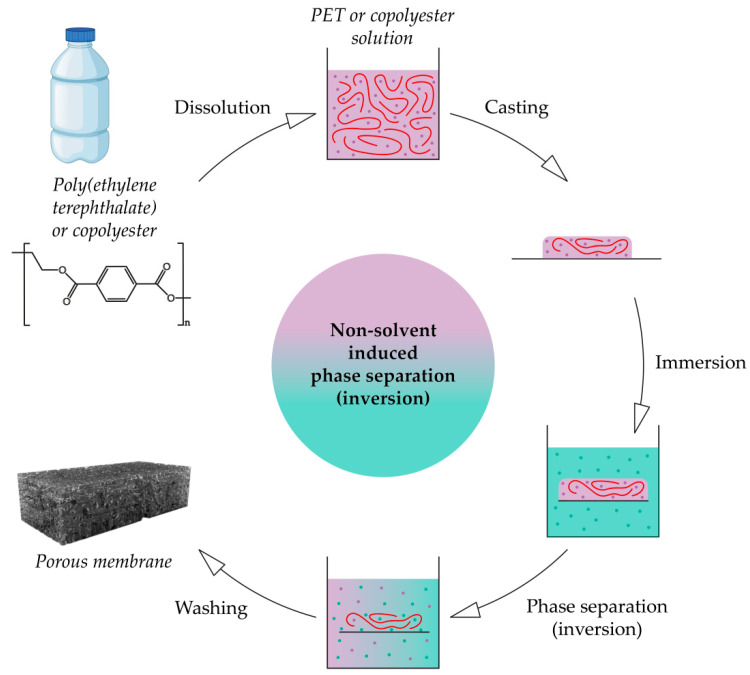
Scheme for obtaining a membrane from rPET or a PET-based copolymer by the non-solvent induced phase separation.

**Figure 9:**
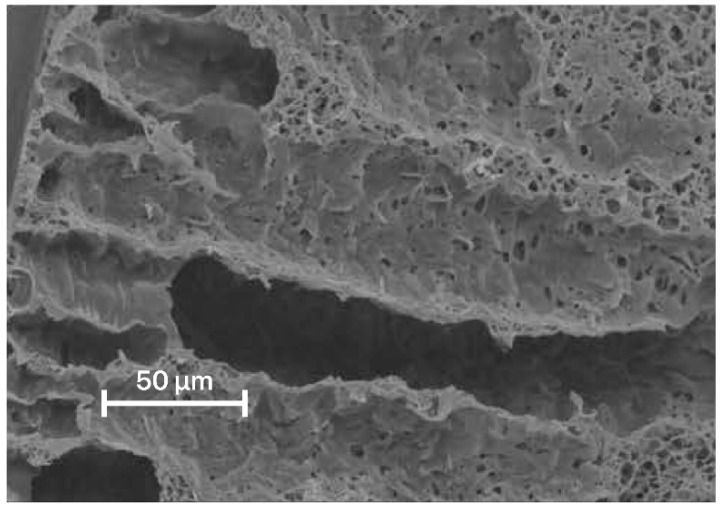
SEM image of a porous (NIPS obtained) membrane [168].

The method of obtaining membranes by phase inversion consists of preparing a polymer solution and removing the solvent from it, resulting in the formation of a porous membrane. Thermally, vapor and non-solvent induced phase separations are distinguished, depending on the solvent removal method [18]. The non-solvent induced phase separation method (NIPS) is most commonly used in the case of PET [158,159,160,161,162,163,164,165,166]. The scheme of the process is shown in Figure 8. The polymer is dissolved in a suitable solvent followed by the formation of a thin film, that is further immersed into the precipitation bath with a non-solvent. When the solution comes into contact with the precipitant, the polymer precipitates, which leads to the formation of a membrane [167]. The membrane thus obtained, is washed thoroughly. The SEM image of a porous (NIPS obtained) membrane is shown in Figure 9.

The choice of the solvent is an important technological factor [158] as in the case of nanofiber membranes. The composition varies in both solvent and non-solvent. Example systems are shown in Table 5.

#### 3.3.1. PET-Based Porous Membranes

Special substances are added to the composition, in addition to solvents to form pores. They are usually combined by mixing a PET solution and an additive solution in the same solvent [165,166]. This additive must be soluble both in the solvent and in the non-solvent, since it is leached from PET during the phase separation step. Water-soluble polymers, such as polyvinyl alcohol and polyvinylpyrrolidone [160], polyethylene glycols [162,165,166] and xanthan gum [161] are used. The main purpose of these polymers is the formation of pores, and their influence on the properties of the resulting membrane is limited only by their influence on the size and distribution of pores.

#### 3.3.2. Copolyester-Based Porous Membranes

Polyesters are used to fabricate porous membranes for various applications, in particular, microporous membrane filters [169] or electrochemical cells [170]. The main objectives of using copolyesters, instead of PET, are the same as those indicated for the nanofiber membranes above, since both are molded from a solution. For example, such a terephthalate-based copolyester may include glycol units with three to seven carbon atoms [169], in particular, butylene glycol or 1,3-propanediol [170]. Polyvinyl alcohol or polyvinyl pyrrolidone [169] and polyethers [170] are used as water-soluble additives. The authors indicate that the advantage of such porous membranes is the absence of a static charge, which leads to dust particles adhering to the membrane [169]. Other advantageous characteristics of the porous polyester membranes are their wettability with water and other electrolytic solvents, mechanical strength, and thermal stability [170].

It is also possible to obtain porous membranes with phase inversion, using unsaturated polyester resins, which can be obtained on the basis of rPET [12,13,14]. For example, Jimenez et al. used unsaturated polyester resins, in combination with natural rubber (latex), dissolving them in dichloromethane and crosslinking under the action of a sulfonating agent [171]. The solvent was removed by evaporation. The authors proposed to use similar proton-exchange membranes for fuel cells.

### 3.4. Other Methods

There are other less common methods for obtaining PET-based membranes, in addition to track etching, electrospinning and phase inversion. One such method is the perforation of PET film with a laser [172]. These membranes could be used for the separation processes and as chemical sensors. Pyrolysis is also common for obtaining porous carbon layers [173,174]. Thus, a carbon membrane for gas purification was obtained [173]. First, the porous substrate was coated with a polyester solution, then the solvent was removed, followed by the pyrolysis of the obtained coating. The authors noted that the use of unsaturated polyesters of various compositions is the most preferable in such processes. The main direction of application of such membranes is gas separation [173].

## 4. Conclusions and Perspectives

The recycling of PET waste is an urgent task for modern science and industry, and membrane technologies are a potentially promising field of application for the obtained materials. Among the currently known four major recycling approaches shown in Section 2: primary (re-extrusion), secondary (mechanical), tertiary (chemical) and incineration, chemical recycling methods have the greatest potential for further development. The main reason for this is the possibility of obtaining PET, close in properties to the virgin one, which can be used without additional restrictions in the food and medical industries. Under such conditions, theoretically, the polymer can be recycled an unlimited number of times without reducing the quality. This seems to be much more valuable than primary and secondary recycling, since in the latter case, the quality of rPET deteriorates in each processing cycle, due to thermal-oxidative degradation and hydrolysis.

It follows from the reviewed works that chemical recycling through glycolysis of rPET to bis(2-hydroxyethyl)terephthalate is the most promising way to obtain pure PET, since glycolysis does not require expensive equipment, additional separation steps, and the product can be easily purified. The process can be organized homogeneously or heterogeneously, depending on the conditions and the agents used. Copolyesters, based on recycled PET, can be obtained in two ways: by degradation of polyethylene terephthalate to monomers or the low molecular weight oligoethylene terephthalates, followed by the polycondensation with the addition of comonomers or by interchain exchange of polyester from comonomers with polyethylene terephthalate. The first method makes it possible to obtain polymers applicable in medicine and the food industry, in the case of the sufficient purification of the obtained monomers. The second method, in turn, is much cheaper, which makes it possible to use the copolyesters obtained in this way for mass technical applications.

The main methods for obtaining membranes, based on polyethylene terephthalate and copolyesters are (1) ion track technology, (2) electrospinning and (3) phase inversion (separation). Other applicable methods for obtaining membranes are the use of a laser and pyrolysis (including for unsaturated polyester resins). Let us summarize the applicability of one or another recycling method for obtaining feedstock in the listed membrane manufacturing processes.

Materials obtained by primary, secondary recycling and all methods of chemical recycling of PET are equally suitable to obtain track membranes, because they are further used for technical purification/filtration purposes and other tasks not directly related to human uses. Electrospinning gives nanofibrous nonwoven membranes, which are mainly used for gas purification. In this case, special requirements are imposed on membranes used in personal protective equipment. Along with the use of virgin PET, source materials of suitable quality could be obtained by degradation-based chemical processing, described in Section 2. At the same time, nanofiber materials can be of any origin for technical applications. Additional modification of membranes made both by ion track technology and electrospinning, is possible to customize their properties or give new ones. The main methods of modification are the grafting of various substances, usually organosilicon compounds and monomers containing multiple bonds, and the application of agents, such as polyelectrolytes, nanoparticles and nanotubes. The main application of porous membranes obtained by phase inversion (phase separation) is water treatment. The information on possible combinations of solvent-non-solvent and the blowing agents was systematized. It is permissible to add non-solvent-soluble polymer additives, for example, polyvinyl alcohol, polyvinylpyrrolidone, polyethylene glycols and xanthan gum. Since the known applications are mostly technical, the purity of rPET is determined solely by the manufacturing process itself.

The main disadvantage of PET chemical recycling technologies is their high cost, which limits their wide application in industry. The cost is determined by the duration of the processes and the need to use catalysts and high temperatures. However, these technologies can be applied as upcycling technologies to obtain products with a high added value, including various types of membranes. An urgent task for the development of technologies for obtaining membranes is the search for new ways of modifying surfaces and agents used for this modification. Thus, promising directions for further research on recycled PET-based membranes are the improvement of technologies for the recycling of PET, the production of membranes, based on it by electrospinning and phase separation, and the post-processing of track and nanofiber membranes. The study of methods for obtaining copolyesters, based on rPET and the properties of membranes, based on them, is also a potentially significant direction. PET-based copolyesters that are highly soluble in organic solvents can be of particular value for electrospinning and non-solvent induced phase separation technologies.

## Figures and Tables

**Table 1:** Disadvantages of various recycling methods.

Disadvantage	Primary and Secondary Recycling	Incineration	Tertiary (Chemical) Recycling
Deterioration of polymer properties	+	+	-
Moisture sensitivity	+	-	-
Negative effect of additives on properties	+	-	+/- ^1^
Low process rate	-	-	+
The need to regenerate liquid components	-	-	+
Toxicity of the components	-	+	+

^1^ Additives, in particular dyes, can be easily removed during the depolymerization of poly(ethylene terephthalate). Nevertheless, their presence will affect the properties of the interchain exchange copolymers.

**Table 2:** Surface hydrophilization methods.

Method	Agents	References
Grafting	Polyvinyl alcohol, glutaraldehyde (binding agent), hydrochloric acid (catalyst)	[83]
	Acrylic acid, N-vinylimidazole	[84]
Oxidation	Hydrogen peroxide	[84,85]
UV treatment		[86]
Plasma treatment		[86,87]
Steam treatment		[87] ^1^

^1^ Steam sterilization had been found not to increase the hydrophility further.

**Table 3:** Used types of membrane coatings and their properties.

Coating Type	Substances	Properties	Applications	References
Polyelectrolyte	Polyaniline	Electrical conductivity and other electrophysical properties	Detection of the charged molecules	[95,96]
	Polypyrrole			[95]
	Poly(2-acrylamido glycolic acid)			[97]
	Poly(N-acetyl dehydroalanine)			[97]
	Methacrylic acid/poly(allylamine)		Heavy ions detection	[98]
Nanoparticles	Titanium	Electrical conductivity, chemical and thermal stability	Production of the sensitive electrodes	[99,100,101]
	Titanium dioxide	Catalytic activity	Preparation of the catalysts	[99,100,102]
	Cuprous oxide			[103]
	Zinc oxide			[103,104]
	Gold			[105]
	Silver		Detection of acetaminophen in water	[106]
	Co_0·5_ Ni_0·5_ FeCrO_4_	Magnetic properties	Gas adsorption or separation, water purification	[107]
		Adsorption properties		
	Metal–organic framework			[108,109]
	Pd		Hydrogen purification	[110,111]
Tubes and wires	Ag nanowires		SERS analysis	[112]
	Nickel/gold microtubes			[113,114]
	Gold microtubes			[113,114]
		Catalytic activity	Preparation of the catalysts	[115,116,117]
	Silver microtubes			[118,119,120,121]
	Copper microtubes			[122,123,124]
		Adsorption properties	Removal of the arsenic compounds	[125]
	Carbon nanotubes		Gas adsorption or separation, water purification	[107,108]
		Lightweight, electrical conductivity, low specific heat	Production of acoustic membranes, production of batteries, protection against electromagnetic interference	[126]
	Fe/Ni nanotubes		Targeted delivery of drugs and proteins	[127]
	Fe/Co nanotubes			[128]
	Fe nanotubes		Preparation of lithium-ion batteries	[129]

**Table 5:** Examples of membrane formation via the non-solvent induced phase separation.

Component Type	Substances	References
Solvent	Trifluoroacetic acid, dichloromethane	[159,160]
	Trifluoroacetic acid	[161,162]
	1,1,1,3,3,3-Hexafluoro-2-propanol	[162]
	Phenol, 100 °C	[165,166]
	m-Cresol, 100 °C	[165]
	Dimethyl sulfoxide, 100 °C	[165]
Non-solvent	Water	[160,161,162]
	Methanol	[161,162]
	Ethanol	[162,165,166]
	n-Propanol	[165]
	n-Butanol	[165]

## Data Availability

Not applicable.

## References

[1] Watt E., Picard M., Maldonado B., Abdelwahab M.A., Mielewski D.F., Drzal L.T., Misra M., Mohanty A.K. (2021). Ocean Plastics: Environmental Implications and Potential Routes for Mitigation—A Perspective. RSC Adv..

[2] Benyathiar P., Kumar P., Carpenter G., Brace J., Mishra D.K. (2022). Polyethylene Terephthalate (PET) Bottle-to-Bottle Recycling for the Beverage Industry: A Review. Polymers.

[3] Juanga-Labayen J.P., Labayen I.V., Yuan Q. (2022). A Review on Textile Recycling Practices and Challenges. Textiles.

[4] Kirshanov K., Toms R., Melnikov P., Gervald A. (2022). Investigation of Polyester Tire Cord Glycolysis Accompanied by Rubber Crumb Devulcanization. Polymers.

[5] Kirshanov K.A., Toms R.V., Gerval’d A.Y. (2022). Prospects of Polyester Tire Cord Waste Utilization. Kauchuk I Rezina.

[6] Bogusz P., Miedzińska D., Wieczorek M. (2022). Experimental Investigation of the Tensile Behavior of Selected Tire Cords Using Novel Testing Equipment. Materials.

[7] Kirshanov K.A., Gervald A.Y.U., Toms R.V., Balashov M.S. (2020). Natural Latex Deproteinization Issues. Kauchuk I Rezina.

[8] Kirshanov K.A., Gervald A.Y. (2021). Elastomeric Compositions in Wound Dressings. Kauchuk I Rezina.

[9] Burrows S.D., Ribeiro F., O’Brien S., Okoffo E., Toapanta T., Charlton N., Kaserzon S., Lin C.-Y., Tang C., Rauert C. (2022). The Message on the Bottle: Rethinking Plastic Labelling to Better Encourage Sustainable Use. Environ. Sci. Policy.

[10] Kirshanov K.A., Gervald A.Y.U., Toms R.V., Lobanov A.N. (2022). Obtaining Phthalate Substituted Post-Consumer Polyethylene Terephthalate and Its Isothermal Crystallization. Fine Chem. Technol..

[11] Hou D., Xin J., Lu X., Guo X., Dong H., Ren B., Zhang S. (2016). Conversion of Bis(2-Hydroxyethylene Terephthalate) into 1,4-Cyclohexanedimethanol by Selective Hydrogenation Using RuPtSn/Al_2_O_3_. RSC Adv..

[12] Kirshanov K.A., Toms R.V., Gervald A.Y. (2022). Study of Methods for Obtaining Unsaturated Polyester Resins Based on Recycled Polyethylene Terephthalate. Plast. Massy.

[13] Kirshanov K., Toms R., Melnikov P., Gervald A. (2022). Unsaturated Polyester Resin Nanocomposites Based on Post-Consumer Polyethylene Terephthalate. Polymers.

[14] Bórquez-Mendivil A., Hurtado-Macías A., Leal-Pérez J.E., Flores-Valenzuela J., Vargas-Ortíz R.Á., Cabrera-Covarrubias F.G., Almaral-Sánchez J.L. (2022). Hybrid Coatings of SiO2–Recycled PET Unsaturated Polyester Resin by Sol-Gel Process. Polymers.

[15] Li M.-J., Huang Y.-H., Ju A.-Q., Yu T.-S., Ge M.-Q. (2014). Synthesis and Characterization of Azo Dyestuff Based on Bis(2-Hydroxyethyl) Terephthalate Derived from Depolymerized Waste Poly(Ethylene Terephthalate) Fibers. Chin. Chem. Lett..

[16] Kamrani H., Nosrati A. (2018). Fabrication of Nanofiber Filtration Membranes Using Polyethylene Terephthalate (PET): A Review. J. Membr. Sci. Technol.

[17] Al-Shaeli M., Al-Juboori R.A., al Aani S., Ladewig B.P., Hilal N. (2022). Natural and Recycled Materials for Sustainable Membrane Modification: Recent Trends and Prospects. Sci. Total Environ..

[18] Yeszhanov A.B., Korolkov I.V., Dosmagambetova S.S., Zdorovets M.V., Güven O. (2021). Recent Progress in the Membrane Distillation and Impact of Track-Etched Membranes. Polymers.

[19] Al-Sabagh A.M., Yehia F.Z., Eshaq G.H., Rabie A.M., ElMetwally A.E. (2016). Greener Routes for Recycling of Polyethylene Terephthalate. Egypt. J. Pet..

[20] Damayanti, Wu H.-S. (2021). Strategic Possibility Routes of Recycled PET. Polymers.

[21] Suhaimi N.A.S., Muhamad F., Abd Razak N.A., Zeimaran E. (2022). Recycling of Polyethylene Terephthalate Wastes: A Review of Technologies, Routes, and Applications. Polym. Eng. Sci..

[22] Geyer B., Lorenz G., Kandelbauer A. (2016). Recycling of Poly(Ethylene Terephthalate)—A Review Focusing on Chemical Methods. Express Polym. Lett..

[23] Nikles D.E., Farahat M.S. (2005). New Motivation for the Depolymerization Products Derived from Poly(Ethylene Terephthalate) (PET) Waste: A Review. Macromol. Mater. Eng..

[24] Raheem A.B., Noor Z.Z., Hassan A., Abd Hamid M.K., Samsudin S.A., Sabeen A.H. (2019). Current Developments in Chemical Recycling of Post-Consumer Polyethylene Terephthalate Wastes for New Materials Production: A Review. J. Clean. Prod..

[25] Ghosal K., Nayak C. (2022). Recent Advances in Chemical Recycling of Polyethylene Terephthalate Waste into Value Added Products for Sustainable Coating Solutions—Hope vs. Hype. Mater. Adv..

[26] Aguado A., Martínez L., Becerra L., Arieta-araunabeña M., Arnaiz S., Asueta A., Robertson I. (2014). Chemical Depolymerisation of PET Complex Waste: Hydrolysis vs. Glycolysis. J. Mater. Cycles Waste Manag..

[27] Raheem A.B., Hassan A.B., Noor Z.Z., Samsudin S.B., Hamid M.A., Bello A., Oladokun O., Sabee A.H., Shamiri A. (2018). Process Simulation of Bis (2- Hydroxyethyl) Terephthalate and Its Recovery Using Two–Stage Evaporation Systems. Chem. Eng. Trans..

[28] Lu J., Li M., Li Y., Li X., Gao Q., Ge M. (2019). Synthesis and Sizing Performances of Water-Soluble Polyester Based on Bis(2-Hydroxyethyl) Terephthalate Derived from Depolymerized Waste Poly(Ethylene Terephthalate) Fabrics. Text. Res. J..

[29] Liu B., Lu X., Ju Z., Sun P., Xin J., Yao X., Zhou Q., Zhang S. (2018). Ultrafast Homogeneous Glycolysis of Waste Polyethylene Terephthalate via a Dissolution-Degradation Strategy. Ind. Eng. Chem. Res..

[30] Stoski A., Viante M.F., Nunes C.S., Muniz E.C., Felsner M.L., Almeida C.A.P. (2016). Oligomer Production through Glycolysis of Poly(Ethylene Terephthalate): Effects of Temperature and Water Content on Reaction Extent. Polym. Int..

[31] Hoang C.N., Pham C.T., Dang T.M., Hoang D., Lee P.-C., Kang S.-J., Kim J. (2019). Novel Oligo-Ester-Ether-Diol Prepared by Waste Poly(Ethylene Terephthalate) Glycolysis and Its Use in Preparing Thermally Stable and Flame Retardant Polyurethane Foam. Polymers.

[32] Fuentes C.A., Gallegos M.V., García J.R., Sambeth J., Peluso M.A. (2020). Catalytic Glycolysis of Poly(Ethylene Terephthalate) Using Zinc and Cobalt Oxides Recycled from Spent Batteries. Waste Biomass Valorization.

[33] Chen F., Zhou Q., Bu R., Yang F., Li W. (2015). Kinetics of Poly(Ethylene Terephthalate) Fiber Glycolysis in Ethylene Glycol. Fibers Polym..

[34] Scé F., Cano I., Martin C., Beobide G., Castillo Ó., de Pedro I. (2019). Comparing Conventional and Microwave-Assisted Heating in PET Degradation Mediated by Imidazolium-Based Halometallate Complexes. New J. Chem..

[35] Esquer R., García J.J. (2019). Metal-Catalysed Poly(Ethylene) Terephthalate and Polyurethane Degradations by Glycolysis. J. Organomet. Chem..

[36] Fang P., Liu B., Xu J., Zhou Q., Zhang S., Ma J., Lu X. (2018). High-Efficiency Glycolysis of Poly(Ethylene Terephthalate) by Sandwich-Structure Polyoxometalate Catalyst with Two Active Sites. Polym. Degrad. Stab..

[37] Li M., Li Y., Lu J., Li X., Lu Y. (2018). Decolorization and Reusing of PET Depolymerization Waste Liquid by Electrochemical Method with Magnetic Nanoelectrodes. Environ. Sci. Pollut. Res..

[38] Nabid M.R., Bide Y., Jafari M. (2019). Boron Nitride Nanosheets Decorated with Fe3O4 Nanoparticles as a Magnetic Bifunctional Catalyst for Post-Consumer PET Wastes Recycling. Polym. Degrad. Stab..

[39] Alzuhairi M. (2018). Bubble Column and CFD Simulation for Chemical Recycling of Polyethylene Terephthalate. AIP Conference Proceedings.

[40] Cano I., Martin C., Fernandes J.A., Lodge R.W., Dupont J., Casado-Carmona F.A., Lucena R., Cardenas S., Sans V., de Pedro I. (2020). Paramagnetic Ionic Liquid-Coated SiO_2_@Fe_3_O_4_ Nanoparticles—The next Generation of Magnetically Recoverable Nanocatalysts Applied in the Glycolysis of PET. Appl. Catal. B.

[41] Zhao Y., Liu M., Zhao R., Liu F., Ge X., Yu S. (2018). Heterogeneous CaO(SrO, BaO)/MCF as Highly Active and Recyclable Catalysts for the Glycolysis of Poly(Ethylene Terephthalate). Res. Chem. Intermed..

[42] Park G., Bartolome L., Lee K.G., Lee S.J., Kim D.H., Park T.J. (2012). One-Step Sonochemical Synthesis of a Graphene Oxide–Manganese Oxide Nanocomposite for Catalytic Glycolysis of Poly(Ethylene Terephthalate). Nanoscale.

[43] Guo Z., Lindqvist K., de la Motte H. (2018). An Efficient Recycling Process of Glycolysis of PET in the Presence of a Sustainable Nanocatalyst. J. Appl. Polym. Sci..

[44] Veregue F.R., Pereira da Silva C.T., Moisés M.P., Meneguin J.G., Guilherme M.R., Arroyo P.A., Favaro S.L., Radovanovic E., Girotto E.M., Rinaldi A.W. (2018). Ultrasmall Cobalt Nanoparticles as a Catalyst for PET Glycolysis: A Green Protocol for Pure Hydroxyethyl Terephthalate Precipitation without Water. ACS Sustain. Chem. Eng..

[45] Lima G.R., Monteiro W.F., Ligabue R., Santana R.M.C. (2017). Titanate Nanotubes as New Nanostrutured Catalyst for Depolymerization of PET by Glycolysis Reaction. Mater. Res..

[46] Lima G.R., Monteiro W.F., Toledo B.O., Ligabue R.A., Santana R.M.C. (2019). Titanate Nanotubes Modified with Zinc and Its Application in Post-Consumer PET Depolymerization. Macromol. Symp..

[47] Al-Sabagh A.M., Yehia F.Z., Harding D.R.K., Eshaq G.H., ElMetwally A.E. (2016). Fe_3_O_4_-Boosted MWCNT as an Efficient Sustainable Catalyst for PET Glycolysis. Green Chem..

[48] Al-Sabagh A.M., Yehia F.Z., Eshaq G.H., ElMetwally A.E. (2015). Ionic Liquid-Coordinated Ferrous Acetate Complex Immobilized on Bentonite as a Novel Separable Catalyst for PET Glycolysis. Ind. Eng. Chem. Res..

[49] Sert E., Yılmaz E., Atalay F.S. (2019). Chemical Recycling of Polyethlylene Terephthalate by Glycolysis Using Deep Eutectic Solvents. J. Polym. Environ..

[50] Liu B., Fu W., Lu X., Zhou Q., Zhang S. (2019). Lewis Acid–Base Synergistic Catalysis for Polyethylene Terephthalate Degradation by 1,3-Dimethylurea/Zn(OAc)_2_ Deep Eutectic Solvent. ACS Sustain. Chem. Eng..

[51] Yunita I., Putisompon S., Chumkaeo P., Poonsawat T., Somsook E. (2019). Effective Catalysts Derived from Waste Ostrich Eggshells for Glycolysis of Post-Consumer PET Bottles. Chem. Pap..

[52] Wang Q., Yao X., Tang S., Lu X., Zhang X., Zhang S. (2012). Urea as an Efficient and Reusable Catalyst for the Glycolysis of Poly(Ethylene Terephthalate) Wastes and the Role of Hydrogen Bond in This Process. Green Chem..

[53] De Castro A.M., Carniel A. (2017). A Novel Process for Poly(Ethylene Terephthalate) Depolymerization via Enzyme-Catalyzed Glycolysis. Biochem. Eng. J..

[54] Jamdar V., Kathalewar M., Jagtap R.N., Dubey K.A., Sabnis A. (2015). Effect of γ-Irradiation on Glycolysis of PET Waste and Preparation of Ecofriendly Coatings Using Bio-Based and Recycled Materials. Polym. Eng. Sci..

[55] Kirshanov K.A., Gerval’d A.Y., Toms R.V. (2020). Obtaining Oligoesters by Directed Glycolytic Destruction of Polyethylene Terephthalate Waste. Plast. Massy.

[56] Kirshanov K.A., Toms R.V. (2021). Study of Polyethylene Terephthalate Glycolysis with a Mixture of Bis(2-Hydroxyethyl) Terephthalate and Its Oligomers. Plast. Massy.

[57] El Mejjatti A., Harit T., Riahi A., Khiari R., Bouabdallah I., Malek F. (2014). Chemical Recycling of Poly(Ethylene Terephthalate). Application to the Synthesis of Multiblock Copolyesters. Express Polym. Lett..

[58] Nagahata R., Sugiyama J., Velmathi S., Nakao Y., Goto M., Takeuchi K. (2004). Synthesis of Poly(Ethylene Terephthalate-Co-Isophthalate) by Copolymerization of Ethylene Isophthalate Cyclic Dimer and Bis(2-Hydroxyethyl) Terephthalate. Polym. J..

[59] Sun L., Zhang Y., Wang J., Liu F., Jia Z., Liu X., Zhu J. (2019). 2,5-Furandicarboxylic Acid as a Sustainable Alternative to Isophthalic Acid for Synthesis of Amorphous Poly(Ethylene Terephthalate) Copolyester with Enhanced Performance. J. Appl. Polym. Sci..

[60] Gan Z., Qu S., Li S., Tan T., Yang J. (2021). Facile Synthesis of PET-Based Poly(Ether Ester)s with Striking Physical and Mechanical Properties. React. Funct. Polym..

[61] Shirali H., Rafizadeh M., Taromi F.A. (2014). Synthesis and Characterization of Amorphous and Impermeable Poly(Ethylene-Co-1,4-Cyclohexylenedimethylene Terephthalate)/Organoclay Nanocomposite via in Situ Polymerization. J. Compos. Mater..

[62] Kim J.H., Lee S.Y., Park J.H., Lyoo W.S., Noh S.K. (2000). Kinetics of Polycondensation and Copolycondensation of Bis(3-Hydroxypropyl Terephthalate) and Bis(2-Hydroxyethyl Terephthalate). J. Appl. Polym. Sci..

[63] Lewis C.L., Spruiell J.E. (2006). Crystallization of 2-Methyl-1,3-Propanediol Substituted Poly(Ethylene Terephthalate). I. Thermal Behavior and Isothermal Crystallization. J. Appl. Polym. Sci..

[64] Tsai Y., Fan C.-H., Wu J.-H. (2016). Synthesis, Microstructures and Properties of Amorphous Poly(Ethylene Terephthalate-Co- Tricyclodecanedimethylene Terephthalate). J. Polym. Res..

[65] Heidarzadeh N., Rafizadeh M., Taromi F.A., del Valle L.J., Franco L., Puiggalí J. (2017). Biodegradability and Biocompatibility of Copoly(Butylene Sebacate-Co-Terephthalate)s. Polym. Degrad. Stab..

[66] Collins S., Peace S.K., Richards R.W., MacDonald W.A., Mills P., King S.M. (2000). Transesterification in Poly(Ethylene Terephthalate). Molecular Weight and End Group Effects. Macromolecules.

[67] Yeszhanov A.B., Korolkov I.V., Gorin Y.G., Dosmagambetova S.S., Zdorovets M.V. (2020). Membrane Distillation of Pesticide Solutions Using Hydrophobic Track-Etched Membranes. Chem. Pap..

[68] Kozhina E., Kulesh E., Bedin S., Doludenko I., Piryazev A., Korolkov I., Kozlovskiy A., Zdorovets M., Rogachev A., Shumskaya A. (2022). One-Dimensional Magneto-Optical Nanostructures: Template Synthesis, Structure, Properties, and Application in Spectroscopy Based on Plasmon Resonance. IEEE Magn. Lett..

[69] Kovalets N.P., Panov D.V., Filippova Y.U.A., Razumovskaya I.V. (2021). Point Agglomeration of Nickel and Iron Nanowires Synthesized in the Pores of Track Membranes. Bull. Russ. Acad. Sci. Phys..

[70] Doludenko I.M., Volchkov I.S., Turenko B.A., Koshelev I.O., Podkur P.L., Zagorskiy D.L., Kanevskii V.M. (2022). Electrical Properties Arrays of Intersecting of Nanowires Obtained in the Pores of Track Membranes. Mater. Chem. Phys..

[71] Doludenko I.M. (2022). Aspects of Pore Filling in Synthesis of FeNi Alloy Nanowires Using Track-Etched Membranes. Inorg. Mater. Appl. Res..

[72] Lee P.L.J., Thangavel V., Guery C., Trautmann C., Toimil-Molares M.E., Morcrette M. (2021). Etched Ion-Track Membranes as Tailored Separators in Li–S Batteries. Nanotechnology.

[73] Temnov D., Rossouw A., Vinogradov I., Shabanova N., Mamonova T., Lizunov N., Perold W., Nechaev A. (2022). Thermo-Activation Spectroscopy of Track-Etched Membranes Based on Polyethylene Terephthalate Films Irradiated by Swift Xe Ions. Radiat. Phys. Chem..

[74] Sokhoreva V.V., Kanaev V.G., Kashkarov E.B., Kulyukina E.S., Kuznetsov S.I. (2018). Formation of a Track Template during PETP Irradiation with High-Energy Helium Ions for the Template Synthesis of Regular Microstructures. J. Surf. Investig. X-ray Synchrotron Neutron Tech..

[75] Apel P.Y.U., Blonskaya I.V., Ivanov O.M., Kristavchuk O.V., Lizunov N.E., Nechaev A.N., Orelovich O.L., Polezhaeva O.A., Dmitriev S.N. (2020). Creation of Ion-Selective Membranes from Polyethylene Terephthalate Films Irradiated with Heavy Ions: Critical Parameters of the Process. Membr. Membr. Technol..

[76] Negi S. (2021). Photo Driven Ion Transport and Pumping through Synthetic Nanochannels. Mater. Today Commun..

[77] Apel P.Y., Blonskaya I.V., Ivanov O.M., Kristavchuk O.V., Nechaev A.N., Olejniczak K., Orelovich O.L., Polezhaeva O.A., Dmitriev S.N. (2022). Do the Soft-Etched and UV-Track Membranes Actually Have Uniform Cylindrical Subnanometer Channels?. Radiat. Phys. Chem..

[78] Zhao J., Du G., Yao H., Guo J., Mao G., Liu W., Wu R., Shen C., Mou H., Zhao C. (2022). Fabrication of Double Conical PET Nanochannel for Molecular Detection. Vacuum.

[79] Wang P., Wang X., Ling Y., Wang M., Ding S., She W., Wang Z., Wang Y., Liu F. (2018). Ultrafast Selective Ionic Transport through Heat-Treated Polyethylene Terephthalate Track Membranes with Sub-Nanometer Pores. Radiat. Meas..

[80] Korolkov I.V., Yeszhanov A.B., Gorin Y.G., Zdorovets M.V., Khlebnikov N.A., Serkov K.V. (2018). Hydrophobization of PET Track-Etched Membranes for Direct Contact Membrane Distillation. Mater. Res. Express.

[81] Korolkov I.V., Narmukhamedova A.R., Melnikova G.B., Muslimova I.B., Yeszhanov A.B., Zhatkanbayeva Z.K., Chizhik S.A., Zdorovets M.V. (2021). Preparation of Hydrophobic PET Track-Etched Membranes for Separation of Oil–Water Emulsion. Membranes.

[82] Korolkov I.V., Gorin Y.G., Yeszhanov A.B., Kozlovskiy A.L., Zdorovets M.V. (2018). Preparation of PET Track-Etched Membranes for Membrane Distillation by Photo-Induced Graft Polymerization. Mater. Chem. Phys..

[83] Shumskaya A., Kaniukov E., Yakimchuk D., Plisko T., Burts K., Bildyukevich A., Nikolaevich L., Kozlovskiy A., Zdorovets M. (2019). Modified Ion-Track Membranes for Separation of Biological Objects. Mater. Res. Express.

[84] Korolkov I.V., Mashentseva A.A., Güven O., Gorin Y.G., Zdorovets M.V. (2018). Protein Fouling of Modified Microporous PET Track-Etched Membranes. Radiat. Phys. Chem..

[85] Mashentseva A.A. (2019). Effect of the Oxidative Modification and Activation of Templates Based on Poly(Ethylene Terephthalate) Track-Etched Membranes on the Electroless Deposition of Copper and the Catalytic Properties of Composite Membranes. Pet. Chem..

[86] Vo T.S., Vo T.T.B.C. (2022). Surface Characterization of Polyimide and Polyethylene Terephthalate Membranes toward Plasma and UV Treatments. Prog. Nat. Sci. Mater. Int..

[87] Filippova E.O., Karpov D.A., Pichugin V.F., Ulbricht M. (2020). The Investigation of the Influence of Low-Temperature Plasma and Steam Sterilization on the Properties of Track Membranes Made of Polyethylene Terephthalate. Inorg. Mater. Appl. Res..

[88] Korolkov I.V., Yeszhanov A.B., Shakayeva A.K., Shlimas D.I., Zhumazhanova A., Zdorovets M.V. (2022). Photo-Induced Graft (Co)Polymerization of Glycidyl Methacrylate and Acrylonitrile on PET Ion-Track Membranes for Electrochemical Detection of Uranyl Ions. Colloids Surf. A Physicochem. Eng. Asp..

[89] Zdorovets M.V., Korolkov I.V., Yeszhanov A.B., Gorin Y.G. (2019). Functionalization of PET Track-Etched Membranes by UV-Induced Graft (Co)Polymerization for Detection of Heavy Metal Ions in Water. Polymers.

[90] Nguyen T., Jung S.H., Lee M.S., Park T.-E., Ahn S., Kang J.H. (2019). Robust Chemical Bonding of PMMA Microfluidic Devices to Porous PETE Membranes for Reliable Cytotoxicity Testing of Drugs. Lab. Chip..

[91] Parmanbek N., Sütekin D.S., Barsbay M., Mashentseva A.A., Zheltov D.A., Aimanova N.A., Jakupova Z.Y., Zdorovets M.V. (2022). Hybrid PET Track-Etched Membranes Grafted by Well-Defined Poly(2-(Dimethylamino)Ethyl Methacrylate) Brushes and Loaded with Silver Nanoparticles for the Removal of As(III). Polymers.

[92] Shang Y., Zhang Y., Li P., Lai J., Kong X.-Y., Liu W., Xiao K., Xie G., Tian Y., Wen L. (2015). DNAzyme Tunable Lead (ii) Gating Based on Ion-Track Etched Conical Nanochannels. Chem. Commun..

[93] Muench F., Hashmi S. (2022). Direct Surface Functionalization with Metal and Metal Oxide Nanostructures. Reference Module in Materials Science and Materials Engineering.

[94] Muench F. (2021). Electroless Plating of Metal Nanomaterials. ChemElectroChem.

[95] Do Nascimento K.T.O., Ratkovski G.P., Pedro G.D.C., Gorza F.D.S., da Silva R.J., de Melo C.P. (2021). Intrinsically Conductive Polymers Hybrid Bilayer Films for the Fluorescence Molecular Diagnosis of the Zika Virus. Colloids Surf. B Biointerfaces.

[96] Hu Y.-L., Hua Y., Pan Z.-Q., Qian J.-H., Yu X.-Y., Bao N., Huo X.-L., Wu Z.-Q., Xia X.-H. (2022). PNP Nanofluidic Transistor with Actively Tunable Current Response and Ionic Signal Amplification. Nano Lett..

[97] Wiedenhöft L., Elleithy M.M.A., Ulbricht M., Schacher F.H. (2021). Polyelectrolyte Functionalisation of Track Etched Membranes: Towards Charge-Tuneable Adsorber Materials. Membranes.

[98] Korolkov I.V., Zhumanazar N., Gorin Y.G., Yeszhanov A.B., Zdorovets M.V. (2020). Enhancement of Electrochemical Detection of Pb^2+^ by Sensor Based on Track-Etched Membranes Modified with Interpolyelectrolyte Complexes. J. Mater. Sci. Mater. Electron..

[99] Rossouw A., Olejniczak A., Olejniczak K., Gorberg B., Vinogradov I., Kristavchuk O., Nechaev A., Petrik L., Perold W., Dmitriev S. (2022). Ti and TiO_2_ Magnetron Sputtering in Roll-to-Roll Fabrication of Hybrid Membranes. Surf. Interfaces.

[100] Rossouw A., Kristavchuk O., Olejniczak A., Bode-Aluko C., Gorberg B., Nechaev A., Petrik L., Perold W., Apel P. (2021). Modification of Polyethylene Terephthalate Track Etched Membranes by Planar Magnetron Sputtered Ti/TiO_2_ Thin Films. Thin Solid Films.

[101] Pereao O., Uche C., Bublikov P.S., Bode-Aluko C., Rossouw A., Vinogradov I.I., Nechaev A.N., Opeolu B., Petrik L. (2021). Chitosan/PEO Nanofibers Electrospun on Metallized Track-Etched Membranes: Fabrication and Characterization. Mater. Today Chem..

[102] Kutuzau M., Shumskaya A., Kaniukov E., Alisienok O., Shidlouskaya V., Melnikova G., Shemukhin A., Nazarov A., Kozlovskiy A., Zdorovets M. (2019). Photocatalytically Active Filtration Systems Based on Modified with Titanium Dioxide PET-Membranes. Nucl. Instrum. Methods Phys. Res. B.

[103] Altynbaeva L.S.H., Barsbay M., Aimanova N.A., Jakupova Z.Y., Nurpeisova D.T., Zdorovets M.V., Mashentseva A.A. (2022). A Novel Cu_2_O/ZnO@PET Composite Membrane for the Photocatalytic Degradation of Carbendazim. Nanomaterials.

[104] Khashij M., Salmani M.H., Dalvand A., Fallahzadeh H., Haghirosadat F., Mokhtari M. (2022). Fabrication of ZnO/y-FeOOH Nanoparticles Embedded on the Polyethylene Terephthalate Membrane: Evaluation of Antifouling Behavior and COD Removal. Environ. Sci. Pollut. Res..

[105] Korolkov I.V., Mashentseva A.A., Güven O., Gorin Y.G., Kozlovskiy A.L., Zdorovets M.V., Zhidkov I.S., Cholach S.O. (2018). Electron/Gamma Radiation-Induced Synthesis and Catalytic Activity of Gold Nanoparticles Supported on Track-Etched Poly(Ethylene Terephthalate) Membranes. Mater. Chem. Phys..

[106] Ndilowe G.M., Bode-Aluko C.A., Chimponda D., Kristavchuk O., Kochnev I., Nechaev A., Petrik L. (2021). Fabrication of Silver-Coated PET Track-Etched Membrane as SERS Platform for Detection of Acetaminophen. Colloid Polym. Sci..

[107] Ashtiani S., Khoshnamvand M., Shaliutina-Kolešová A., Bouša D., Sofer Z., Friess K. (2020). Co0·5Ni0·5FeCrO_4_ Spinel Nanoparticles Decorated with UiO-66-Based Metal-Organic Frameworks Grafted onto GO and O-SWCNT for Gas Adsorption and Water Purification. Chemosphere.

[108] Ashtiani S., Sofer Z., Průša F., Friess K. (2021). Molecular-Level Fabrication of Highly Selective Composite ZIF-8-CNT-PDMS Membranes for Effective CO_2_/N_2_, CO_2_/H_2_ and Olefin/Paraffin Separations. Sep. Purif. Technol..

[109] Usman M., Ali M., Al-Maythalony B.A., Ghanem A.S., Saadi O.W., Ali M., Jafar Mazumder M.A., Abdel-Azeim S., Habib M.A., Yamani Z.H. (2020). Highly Efficient Permeation and Separation of Gases with Metal–Organic Frameworks Confined in Polymeric Nanochannels. ACS Appl. Mater. Interfaces.

[110] Awasthi K., Choudhury S., Komber H., Simon F., Formanek P., Sharma A., Stamm M. (2014). Functionalization of Track-Etched Poly (Ethylene Terephthalate) Membranes as a Selective Filter for Hydrogen Purification. Int. J. Hydrogen Energy.

[111] Kamakshi K.R., Saraswat V.K., Kumar M., Awasthi K. (2017). Palladium Nanoparticle Binding in Functionalized Track Etched PET Membrane for Hydrogen Gas Separation. Int. J. Hydrogen Energy.

[112] Kozhina E.P., Bedin S.A., Nechaeva N.L., Podoynitsyn S.N., Tarakanov V.P., Andreev S.N., Grigoriev Y.V., Naumov A.V. (2021). Ag-Nanowire Bundles with Gap Hot Spots Synthesized in Track-Etched Membranes as Effective SERS-Substrates. Appl. Sci..

[113] Korolkov I.V., Shumskaya A., Kozlovskiy A.L., Kaliyekperov M.E., Lissovskaya L.I., Zdorovets M.V. (2022). Magnetic-Plasmonic Ni Nanotubes Covered with Gold for Improvement of SERS Analysis. J. Alloys Compd..

[114] Shumskaya A., Kozhina E., Bedin S., Andreev S., Kulesh E., Rogachev A., Yarmolenko M., Korolkov I., Kozlovskiy A., Zdorovets M. (2022). Detection of Polynitro Compounds at Low Concentrations by SERS Using Ni@Au Nanotubes. Chemosensors.

[115] Mashentseva A., Borgekov D., Kislitsin S., Zdorovets M., Migunova A. (2015). Comparative Catalytic Activity of PET Track-Etched Membranes with Embedded Silver and Gold Nanotubes. Nucl. Instrum. Methods Phys. Res. B.

[116] Mashentseva A., Borgekov D., Zdorovets M., Russakova A. (2014). Synthesis, Structure, and Catalytic Activity of Au/Poly(Ethylene Terephthalate) Composites. Acta Phys. Pol. A.

[117] Mashentseva A.A., Korolkov I.V., Yeszhanov A.B., Zdorovets M.V., Russakova A.V. (2019). The Application of Composite Ion Track Membranes with Embedded Gold Nanotubes in the Reaction of Aminomethylation of Acetophenone. Mater. Res. Express.

[118] Mashentseva A.A., Barsbay M., Aimanova N.A., Zdorovets M.V. (2021). Application of Silver-Loaded Composite Track-Etched Membranes for Photocatalytic Decomposition of Methylene Blue under Visible Light. Membranes.

[119] Mashentseva A.A., Zdorovets M.V. (2017). Composites Based on Polyethylene Terephthalate Track-Etched Membranes and Silver as Hydrogen Peroxide Decomposition Catalysts. Pet. Chem..

[120] Muench F., Rauber M., Stegmann C., Lauterbach S., Kunz U., Kleebe H.-J., Ensinger W. (2011). Ligand-Optimized Electroless Synthesis of Silver Nanotubes and Their Activity in the Reduction of 4-Nitrophenol. Nanotechnology.

[121] Borgekov D., Mashentseva A., Kislitsin S., Kozlovskiy A., Russakova A., Zdorovets M. (2015). Temperature Dependent Catalytic Activity of Ag/PET Ion-Track Membranes Composites. Acta Phys. Pol. A.

[122] Mashentseva A.A., Barsbay M., Zdorovets M.V., Zheltov D.A., Güven O. (2020). Cu/CuO Composite Track-Etched Membranes for Catalytic Decomposition of Nitrophenols and Removal of As(III). Nanomaterials.

[123] Mashentseva A.A., Zdorovets M.V. (2019). Catalytic Activity of Composite Track-Etched Membranes Based on Copper Nanotubes in Flow and Static Modes. Pet. Chem..

[124] Mashentseva A.A., Kozlovskiy A.L., Zdorovets M.V. (2018). Influence of Deposition Temperature on the Structure and Catalytic Properties of the Copper Nanotubes Composite Membranes. Mater. Res. Express.

[125] Russakova A.V., Altynbaeva L.S., Barsbay M., Zheltov D.A., Zdorovets M.V., Mashentseva A.A. (2021). Kinetic and Isotherm Study of As(III) Removal from Aqueous Solution by PET Track-Etched Membranes Loaded with Copper Microtubes. Membranes.

[126] Li Y., Ning W., Peng Q., Yang M., Lei D., Guo S., Liu P., Jiang K., He X., Li Y. (2021). Superbroad-Band Actively Tunable Acoustic Metamaterials Driven from Poly (Ethylene Terephthalate)/Carbon Nanotube Nanocomposite Membranes. Nano Res..

[127] Kaniukov E., Shumskaya A., Yakimchuk D., Kozlovskiy A., Korolkov I., Ibragimova M., Zdorovets M., Kadyrzhanov K., Rusakov V., Fadeev M. (2019). FeNi Nanotubes: Perspective Tool for Targeted Delivery. Appl. Nanosci..

[128] Kozlovskiy A., Zdorovets M., Kadyrzhanov K., Korolkov I., Rusakov V., Nikolaevich L., Fesenko O., Budnyk O., Yakimchuk D., Shumskaya A. (2019). FeCo Nanotubes: Possible Tool for Targeted Delivery of Drugs and Proteins. Appl. Nanosci..

[129] Kozlovskiy A.L., Zdorovets M.V., Shumskaya A.E., Kadyrzhanov K.K. (2019). Study of the Applicability of FE Nanotubes as an Anode Material of Lithium-ion Batteries. Prog. Electromagn. Res. M.

[130] Starosta W., Wawszczak D., Sartowska B., Buczkowski M. (1999). Investigations of Heavy Ion Tracks in Polyethylene Naphthalate Films. Radiat. Meas..

[131] Molokanova L.G., Kochnev Y.U.K., Nechaev A.N., Chukova S.N., Apel P.Y.U. (2017). Effect of Ultraviolet Radiation on Polyethylene Naphthalate Films Irradiated with High-Energy Heavy Ions. High Energy Chem..

[132] Ivanova N.M., Filippova E.O., Tverdokhlebov S.I., Levkovich N.V., Apel P.Y.U. (2021). Preparation, Structure, and Properties of Track-Etched Membranes Based on Polylactic Acid. Membr. Membr. Technol..

[133] Kang H.J., Youm J.S., Kim J.H. (2011). Characteristics of PET-PEN Copolymer as a Material for Flexible Substrate. Polym. Korea.

[134] Krentsel’ L.B., Makarova V.V., Kudryavtsev Y.A.V., Govorun E.N., Litmanovich A.D., Markova G.D., Vasnev V.A., Kulichikhin V.G. (2009). Interchain Exchange and Interdiffusion in Blends of Poly(Ethylene Terephthalate) and Poly(Ethylene Naphthalate). Polym. Sci. Ser. A.

[135] Gunes K., Isayev A.I., Li X., Wesdemiotis C. (2010). Fast in Situ Copolymerization of PET/PEN Blends by Ultrasonically-Aided Extrusion. Polymer.

[136] Buasri A., Ongmali D., Sriboonpeng P., Prompanut S., Loryuenyong V. (2018). Synthesis of PET-PLA Copolymer from Recycle Plastic Bottle and Study of Its Applications in the Electrochromic Devices with Graphene Conductive Ink. Mater. Today Proc..

[137] Flores I., Etxeberria A., Irusta L., Calafel I., Vega J.F., Martínez-Salazar J., Sardon H., Müller A.J. (2019). PET-PLA Partially Degradable Random Copolymers Prepared by Organocatalysis: Effect of Poly( Lactic Acid) Incorporation on Crystallization and Morphology. ACS Sustain. Chem. Eng..

[138] Yasin S., Bakr Z.H., Ali G.A.M., Saeed I. (2021). Recycling Nanofibers from Polyethylene Terephthalate Waste Using Electrospinning Technique. Waste Recycling Technologies for Nanomaterials Manufacturing.

[139] Xu G.-R., An X.-C., Das R., Xu K., Xing Y.-L., Hu Y.-X. (2020). Application of Electrospun Nanofibrous Amphiphobic Membrane Using Low-Cost Poly (Ethylene Terephthalate) for Robust Membrane Distillation. J. Water Process Eng..

[140] Topuz F., Oldal D.G., Szekely G. (2022). Valorization of Polyethylene Terephthalate (PET) Plastic Wastes as Nanofibrous Membranes for Oil Removal: Sustainable Solution for Plastic Waste and Oil Pollution. Ind. Eng. Chem. Res..

[141] Doan H.N., Phong Vo P., Hayashi K., Kinashi K., Sakai W., Tsutsumi N. (2020). Recycled PET as a PDMS-Functionalized Electrospun Fibrous Membrane for Oil-Water Separation. J. Environ. Chem. Eng..

[142] Strain I.N., Wu Q., Pourrahimi A.M., Hedenqvist M.S., Olsson R.T., Andersson R.L. (2015). Electrospinning of Recycled PET to Generate Tough Mesomorphic Fibre Membranes for Smoke Filtration. J. Mater. Chem. A Mater..

[143] Bonfim D.P.F., Cruz F.G.S., Bretas R.E.S., Guerra V.G., Aguiar M.L. (2021). A Sustainable Recycling Alternative: Electrospun PET-Membranes for Air Nanofiltration. Polymers.

[144] Opálková Šišková A., Frajová J., Nosko M. (2020). Recycling of Poly(Ethylene Terephthalate) by Electrospinning to Enhanced the Filtration Efficiency. Mater. Lett..

[145] Opálková Šišková A., Mosnáčková K., Hrůza J., Frajová J., Opálek A., Bučková M., Kozics K., Peer P., Eckstein Andicsová A. (2021). Electrospun Poly(Ethylene Terephthalate)/Silk Fibroin Composite for Filtration Application. Polymers.

[146] Song J., Zhao Q., Meng C., Meng J., Chen Z., Li J. (2021). Hierarchical Porous Recycled PET Nanofibers for High-Efficiency Aerosols and Virus Capturing. ACS Appl. Mater. Interfaces.

[147] Wu H., Zhao H., Lin Y., Liu X., Yao H., Yu L., Wang H., Wang X. (2022). Fabrication of Polysulfone Membrane with Sponge-like Structure by Using Different Non-Woven Fabrics. Sep. Purif. Technol..

[148] Tas M., Musa U.G., Ahmed I., Xu F., Smartt C., Hou X. (2022). Functionalised SiO_2_ Modified Icephobic Nanocomposite Electrospun Membranes for Outdoor Electromagnetic Shielding Applications. Polymer.

[149] Liu R., Qu M., Qiu X., Wang H., Fan M., Zhang A., Chen Q., Bin Y. (2022). Poly (Ethylene Terephthalate) Nonwoven Fabrics-based Membranes Modified by Electrospinning of Thermoplastic Polyurethane, Nano SiO _2_ and Ag Particles as Medical Packing Materials. Packag. Technol. Sci..

[150] Grumezescu A.M., Stoica A.E., Dima-Bălcescu M.-Ș., Chircov C., Gharbia S., Baltă C., Roșu M., Herman H., Holban A.M., Ficai A. (2019). Electrospun Polyethylene Terephthalate Nanofibers Loaded with Silver Nanoparticles: Novel Approach in Anti-Infective Therapy. J. Clin. Med..

[151] De Oliveira Santos R.P., Ramos L.A., Frollini E. (2020). Bio-Based Electrospun Mats Composed of Aligned and Nonaligned Fibers from Cellulose Nanocrystals, Castor Oil, and Recycled PET. Int. J. Biol. Macromol..

[152] Essa W.K., Yasin S.A., Abdullah A.H., Thalji M.R., Saeed I.A., Assiri M.A., Chong K.F., Ali G.A.M. (2022). Taguchi L25 (54) Approach for Methylene Blue Removal by Polyethylene Terephthalate Nanofiber-Multi-Walled Carbon Nanotube Composite. Water.

[153] Gün Gök Z., İnal M., Bozkaya O., Yiğitoğlu M., Vargel İ. (2020). Production of 2-hydroxyethyl Methacrylate-*g*-poly(Ethylene Terephthalate) Nanofibers by Electrospinning and Evaluation of the Properties of the Obtained Nanofibers. J. Appl. Polym. Sci..

[154] Wu C.-S., Wu D.-Y., Wang S.-S. (2022). Characterization and Functionality of Nanocomposite Mats Containing Polyester, Seashell, and Silica Aerogel Using an Electrospinning Fabrication Approach. Polym. Bull..

[155] Danti S., Anand S., Azimi B., Milazzo M., Fusco A., Ricci C., Zavagna L., Linari S., Donnarumma G., Lazzeri A. (2021). Chitin Nanofibril Application in Tympanic Membrane Scaffolds to Modulate Inflammatory and Immune Response. Pharmaceutics.

[156] Cho C.-J., Chang Y.-S., Lin Y.-Z., Jiang D.-H., Chen W.-H., Lin W.-Y., Chen C.-W., Rwei S.-P., Kuo C.-C. (2020). Green Electrospun Nanofiber Membranes Filter Prepared from Novel Biomass Thermoplastic Copolyester: Morphologies and Filtration Properties. J. Taiwan Inst. Chem. Eng..

[157] Baig U., Waheed A. (2022). Fabrication of Polypyrrole-Graphitic Carbon Nitride Nanocomposite Containing Hyper-Cross-Linked Polyamide Photoresponsive Membrane with Self-Cleaning Properties for Water Decontamination and Desalination Applications. J. Water Process Eng..

[158] Lu D., Babaniamansour P., Williams A., Opfar K., Nurick P., Escobar I.C. (2022). Fabrication and Evaporation Time Investigation of Water Treatment Membranes Using Green Solvents and Recycled Polyethylene Terephthalate. J. Appl. Polym. Sci..

[159] Cadore Í.R., Ambrosi A., Cardozo N.S.M., Tessaro I.C. (2022). Poly(Ethylene Terephthalate) Phase Inversion Membranes: Thermodynamics and Effects of a Poor Solvent on the Membrane Characteristics. Polym. Eng. Sci..

[160] Cadore Í.R., Ambrosi A., Cardozo N.S.M., Tessaro I.C. (2019). Phase Separation Behavior of Poly(Ethylene Terephthalate)/(Trifluoroacetic Acid/Dichloromethane)/Water System for Wet Phase Inversion Membrane Preparation. J. Appl. Polym. Sc.i.

[161] Kiani S., Mousavi S.M., Bidaki A. (2021). Preparation of Polyethylene Terephthalate/Xanthan Nanofiltration Membranes Using Recycled Bottles for Removal of Diltiazem from Aqueous Solution. J. Clean. Prod..

[162] Pulido B.A., Habboub O.S., Aristizabal S.L., Szekely G., Nunes S.P. (2019). Recycled Poly(Ethylene Terephthalate) for High Temperature Solvent Resistant Membranes. ACS Appl. Polym. Mater..

[163] Park S.-H., Alammar A., Fulop Z., Pulido B.A., Nunes S.P., Szekely G. (2021). Hydrophobic Thin Film Composite Nanofiltration Membranes Derived Solely from Sustainable Sources. Green Chem..

[164] Park S.-H., Yang C., Ayaril N., Szekely G. (2022). Solvent-Resistant Thin-Film Composite Membranes from Biomass-Derived Building Blocks: Chitosan and 2,5-Furandicarboxaldehyde. ACS Sustain. Chem. Eng..

[165] Kusumocahyo S.P., Ambani S.K., Kusumadewi S., Sutanto H., Widiputri D.I., Kartawiria I.S. (2020). Utilization of Used Polyethylene Terephthalate (PET) Bottles for the Development of Ultrafiltration Membrane. J. Environ. Chem. Eng..

[166] Kusumocahyo S.P., Ambani S.K., Marceline S. (2021). Improved Permeate Flux and Rejection of Ultrafiltration Membranes Prepared from Polyethylene Terephthalate (PET) Bottle Waste. Sustain. Environ. Res..

[167] Ashtiani S., Khoshnamvand M., Číhal P., Dendisová M., Randová A., Bouša D., Shaliutina-Kolešová A., Sofer Z., Friess K. (2020). Fabrication of a PVDF Membrane with Tailored Morphology and Properties via Exploring and Computing Its Ternary Phase Diagram for Wastewater Treatment and Gas Separation Applications. RSC Adv..

[168] Zhang Q., Lu X., Zhao L. (2014). Preparation of Polyvinylidene Fluoride (PVDF) Hollow Fiber Hemodialysis Membranes. Membranes.

[169] Kesting R.E. (1976). Microporous Polyester Membranes and Polymer Assisted Phase Inversion Process for Their Manufacture. U.S. Patent.

[170] Gronwald O., Weber M., Muhlbach K. (2015). Process for Producing Microporous Polyester Membranes for Electronic. Applications. Patent.

[171] Realpe Á., Romero K.A., Acevedo M.T. (2015). Síntesis de Membranas de Intercambio Protónico a Partir de Mezcla de Poliéster Insaturado y Látex Natural, Para Su Uso En Celdas de Combustible. Inf. Tecnol..

[172] Wu L., Sancaktar E. (2020). Effect of PET Support Membrane Thickness on Water Permeation Behavior of Thermally Responsive PNIPAM-g-PET Membranes. J. Memb. Sci..

[173] Voss H., Therre J., Kaltenborn N., Richter H., Voigt I. (2012). Process for Producing Carbon Membranes. U.S. Patent.

[174] Efimov M.N., Vasilev A.A., Muratov D.G., Dzidziguri E.L., Sheverdiyev K.A., Karpacheva G.P. (2022). Conversion of Polyethylene Terephthalate Waste in the Presence of Cobalt Compound into Highly-Porous Metal-Carbon Nanocomposite (c-PET-Co). Compos. Commun..

